# Clinical outcomes and revision strategies following failed internal fixation of peri-trochanteric femoral fractures: a retrospective cohort study

**DOI:** 10.3389/fmed.2026.1773816

**Published:** 2026-03-04

**Authors:** Yujie Zhang, Fan Yang, Chaorong Yu, Hanxiao Zhu, Erman Chen, Hang Li, Weixu Li

**Affiliations:** 1 Department of Orthopedic Surgery, The Second Affiliated Hospital, Zhejiang University School of Medicine, Hangzhou, Zhejiang, China; 2 Orthopedics Research Institute of Zhejiang University, Hangzhou, Zhejiang, China; 3 Key Laboratory of Motor System Disease Research and Precision Therapy of Zhejiang Province, Hangzhou, Zhejiang, China; 4 State Key Laboratory of Transvascular Implantation Devices, Hangzhou, Zhejiang, China; 5 Clinical Research Center of Motor System Disease of Zhejiang Province, Hangzhou, Zhejiang, China

**Keywords:** clinical outcome, femoral peri-trochanteric fractures, hip fractures, internal fixation failure, revision surgery

## Abstract

**Background:**

Revision surgery for femoral peri-trochanteric fractures is a complex and high-risk intervention. This study aimed to systematically examine the multifaceted etiologies of primary internal fixation failures, classify them into distinct categories, and develop tailored revision strategies to enhance overall management and optimize clinical outcomes in peri-trochanteric fracture cases.

**Methods:**

We conducted a retrospective study of 141 peri-trochanteric fractures with failed internal fixation surgery between 2013 and 2023. The cohort comprised 87 males and 54 females, with a mean age of 60.34 ± 16.02 years. Of these, 96 were intertrochanteric fractures classified by the Arbeitsgemeinschaft für Osteosynthesefragen/Orthopedic Trauma Association (AO/OTA) system (28 type A1, 20 type A2, and 48 type A3), and 45 were subtrochanteric fractures classified by the Seinheimer system. We conducted an in-depth analysis of failure mechanisms and subsequently categorized the patients into four groups, each with distinct revision procedures.

**Results:**

The average follow-up time was 15.96 ± 5.61 months. The joint replacement group had a significantly higher mean age (72.13 ± 13.00 years) than the revision internal fixation group (*P* < 0.05). The average revision surgery duration was 143.06 ± 57.29 min, with the joint replacement group having significantly shorter operation times (107.50 ± 41.40 min, *P* < 0.01), which were comparable to those of the type I revision group. Intraoperative blood loss averaged 344.26 ± 335.43 ml, with the type I and joint replacement revision group showing the least blood loss (*P* < 0.01). The mean healing duration was 7.08 ± 3.33 months. Harris hip scores improved from 24.78 ± 6.08 pre-operatively to 80.59 ± 4.54 post-operatively. Patients who underwent type IV revisions had significantly lower scores (*P* < 0.05).

**Conclusions:**

We classified peri-trochanteric fracture surgical failures into four distinct categories based on age, presence of infection, integrity of the femoral head and acetabulum, varus deformity, and implant condition. We developed detailed diagnostic and treatment protocols for each category. Adhering to our established protocols, the imaging results and functional scores of all patients were consistently favorable. Our comprehensive treatment strategy can serve as a critical reference for standard revision procedures in the management of peri-trochanteric fractures.

## 1 Introduction

Peri-trochanteric fractures represent the pre-dominant type of hip fracture in elderly individuals, with an incidence that significantly increases with age and bone density deterioration (1). These fractures primarily encompass intertrochanteric and subtrochanteric fractures, characterized by complex load-bearing regions and dependence on optimal biomechanical conditions for successful healing (2). Femoral peri-trochanteric fractures of the hip in the elderly have devastating impacts on function and quality of life (3, 4). The primary therapeutic objective for peri-trochanteric fractures is to restore patient mobility as promptly as possible, thereby minimizing the risk of long-term complications associated with prolonged immobilization, such as pressure ulcers, pneumonia, and deep vein thrombosis (5). Consequently, surgical intervention is recommended for all such fractures unless contraindicated by the patient's overall health status or the inability to tolerate anesthesia (6). The most commonly used surgical strategies for peri-trochanteric fractures include closed reduction and internal fixation using intramedullary nails, femoral proximal plates, and sliding compression hip screws (7–9). Despite significant advancements in internal fixation devices and surgical techniques, which have substantially improved the success rate of surgery for peri-trochanteric fractures, implant failure still occurs in 6%−20% of cases (10). This is particularly evident in patients with unstable peri-trochanteric fractures or those with osteoporosis. Revision surgery is a significant challenge when internal fixation fails. Factors such as soft tissue adhesion, osteoporosis, bone deformities, and the presence of old internal fixation devices can increase the complexity and difficulty of revision surgeries (11). Consequently, patients undergoing a second operation often experience prolonged surgical time, increased blood loss, higher risk of complications, and extended hospital stay (12). The reasons for internal fixation failure in trochanteric fractures include inadequate fracture reduction, compromised blood supply, insufficient stability of the fracture ends, inappropriate choice of internal fixation devices, osteoporosis, and infections, among others (13). Therefore, revision surgeries should be performed with a comprehensive understanding of the underlying causes of failure, and appropriate revision measures should be implemented. Revision strategies include bone grafting, replacement of longer or thicker intramedullary nails, addition of supplementary plates, osteotomy, joint arthroplasty, and debridement, followed by external fixation (14).

In this study, we performed a detailed retrospective analysis of cases of failed internal fixation for peri-trochanteric fractures. Our primary objective was to thoroughly investigate the underlying causes of these failures, classify them into distinct categories, and develop personalized revision strategies to improve the overall management of peri-trochanteric fractures.

## 2 Materials and methods

### 2.1 Patients

This study involved individuals who underwent revision surgery for femoral trochanteric fractures and fulfilled the following inclusion criteria: 1) failed internal fixation of intertrochanteric or subtrochanteric fractures; 2) revision surgery for femoral trochanteric fractures at our institution and regular follow-up; and 3) femoral trochanteric fractures resulting from traumatic injuries. Patients with pathological fractures and those who underwent femoral osteotomy were excluded. The local ethics committee approved this study. All surgical interventions were conducted with strict adherence to the ethical guidelines outlined in the Declaration of Helsinki. Each participant was fully informed about the study objectives and provided written informed consent.

### 2.2 Pre-operative management

All patients underwent pelvic radiography, anteroposterior and lateral radiography of the affected hip, and three-dimensional computed tomography (CT). Lower extremity venous B-ultrasonography and coagulation function tests were performed to detect lower extremity venous thrombosis. Routine blood tests, C-reactive protein (CRP) levels, and erythrocyte sedimentation rate (ESR) tests were performed to detect the presence of infection. patients >60 years old routinely underwent echocardiographic evaluation of cardiac function.

### 2.3 Classification and surgical strategies

**Figure 1:**
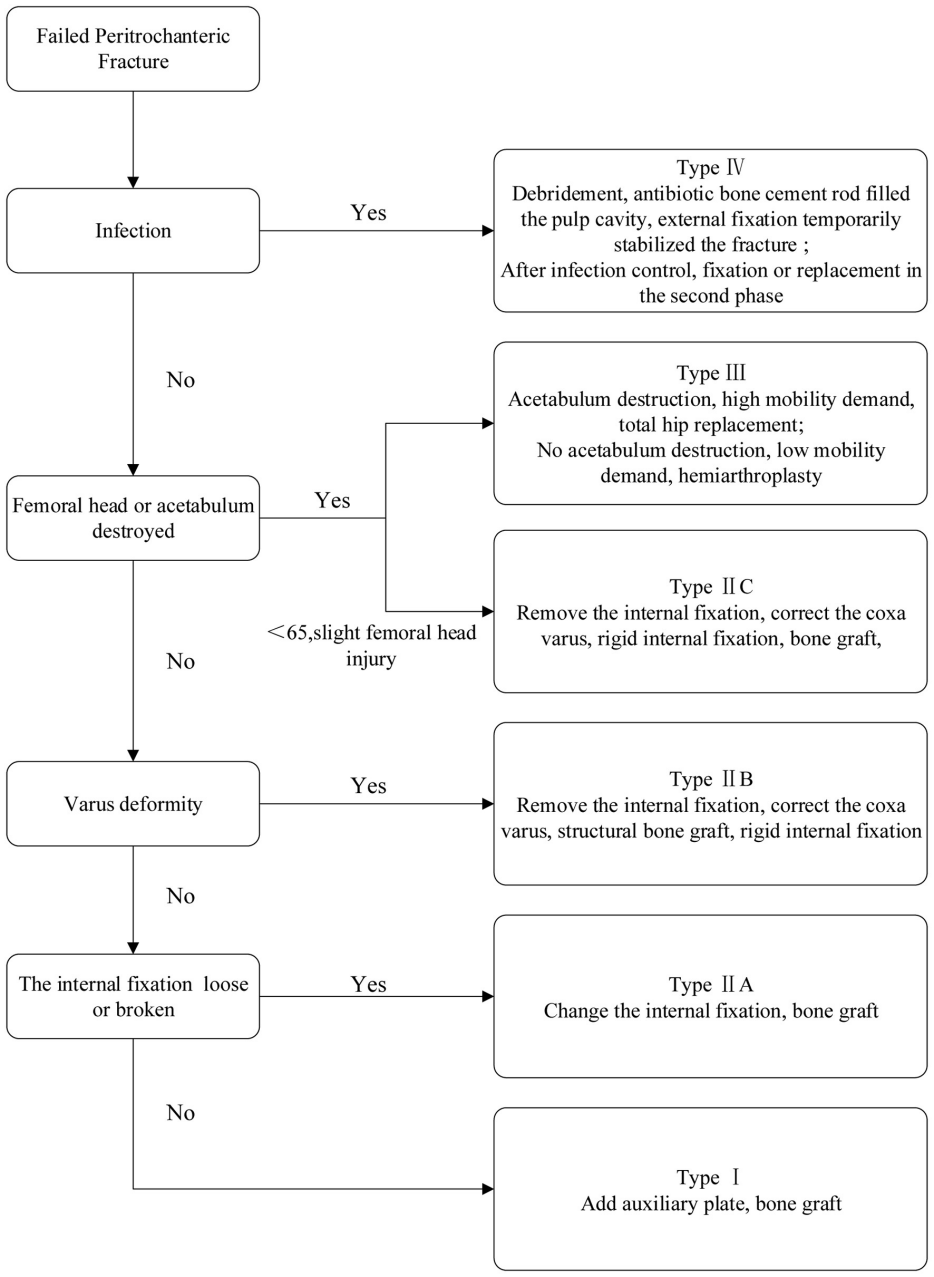
Flowchart for revision strategy following internal fixation failure in femoral peri-trochanteric fractures.

Patients were classified into the following four categories based on the etiology of the initial surgical failure (Figure 1):

I. Non-union with acceptable fracture reduction.
II. A: non-union accompanied by a discontinuity or loosening of the original internal fixation devices. B: non-union with inadequate fracture reduction. C: young patients with screw cutout but minimal femoral head damage.
III. Femoral head resection or necrosis.
IV. Infection.

**Figure 2:**
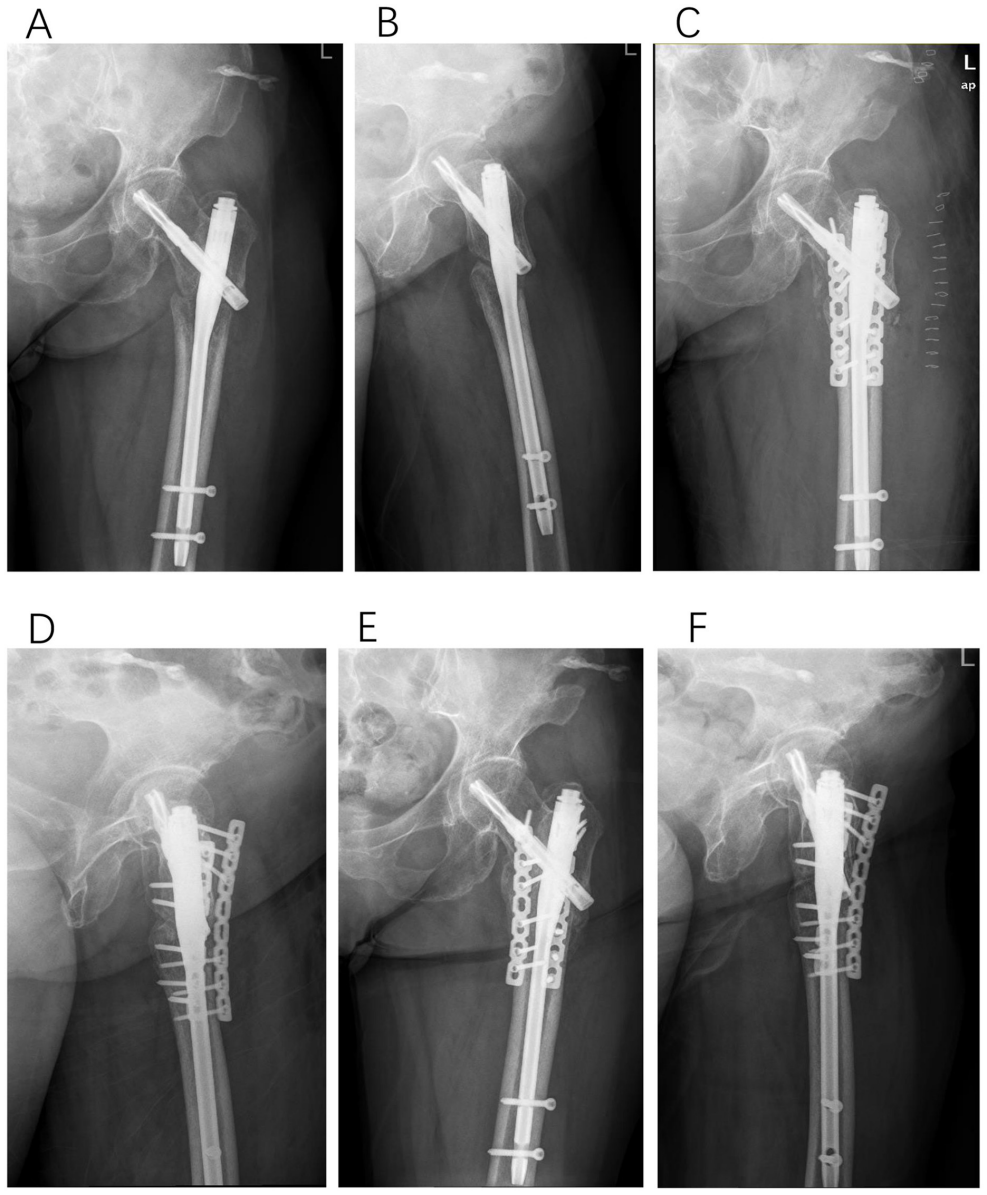
Type I, a 67-year-old female patient underwent PFNA fixation for a left intertrochanteric femoral fracture. Eight months post-operatively, radiographic evaluation revealed non-union at the fracture site. In the revision surgery, two auxiliary plates were added in conjunction with autologous bone grafting. Six months following this revision, successful bony union was achieved. **(A, B)** Anteroposterior and lateral X-rays of the femur obtained 8 months after initial PFNA fixation. **(C, D)** Anteroposterior and lateral X-rays of the femur immediately following the revision surgery. **(E, F)** Successful bony union confirmed 6 months post-revision surgery.

**Figure 3:**
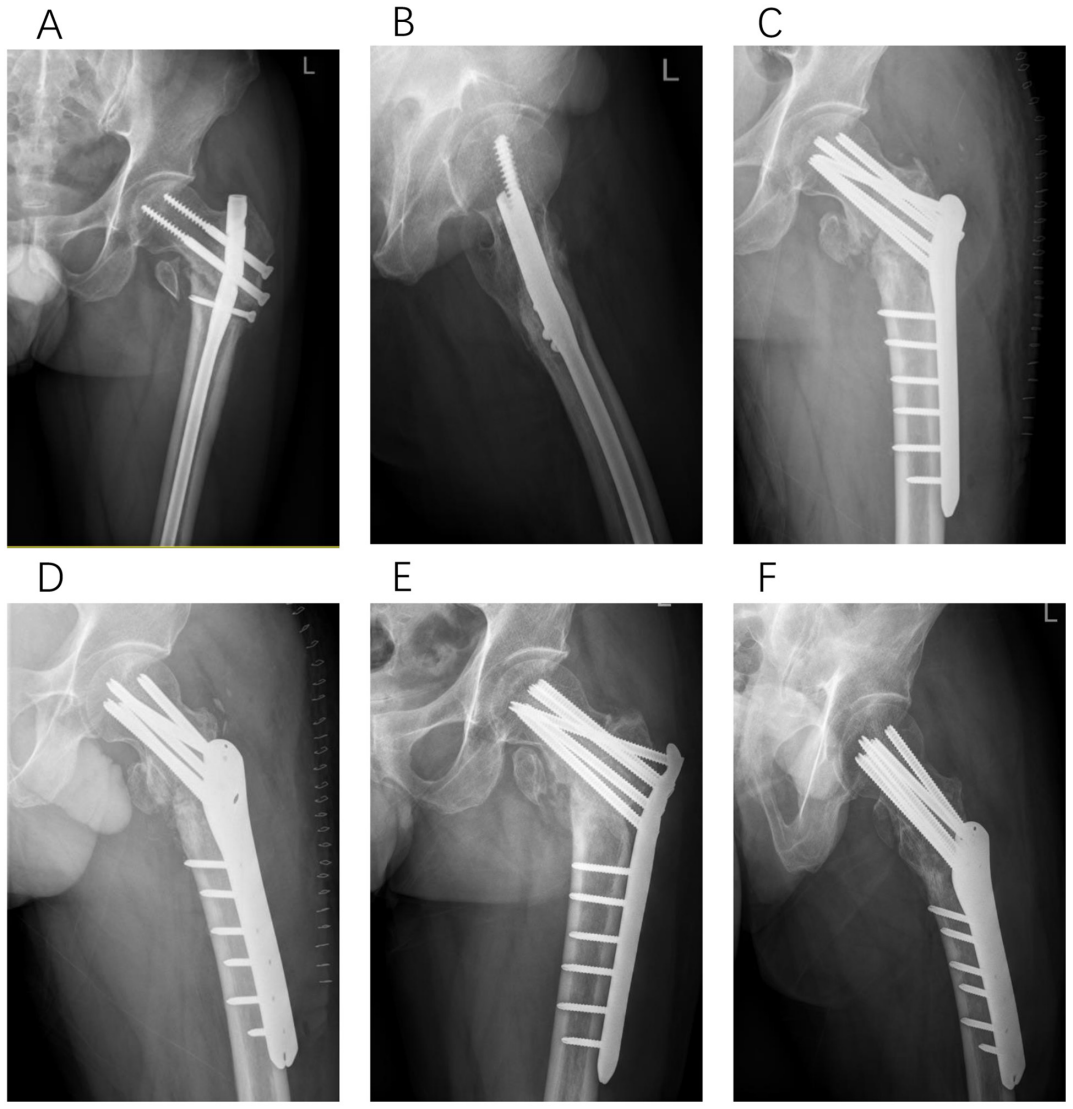
Type II-A, a 51-year-old male patient underwent femoral interlocking nail fixation for a left intertrochanteric femoral fracture. More than 12 months post-operatively, radiographic evaluation revealed the intramedullary nail was broken. In the subsequent revision surgery, we removed the broken nail, corrected the varus deformity via osteotomy, and implemented plate internal fixation. Successful bony union was achieved 3 months following this revision. **(A, B)** Anteroposterior and lateral X-rays of the femur obtained 12 months after initial femoral interlocking nail fixation. **(C, D)** Anteroposterior and lateral X-rays of the femur immediately following the revision surgery. **(E, F)** Successful bony union confirmed 3 months post-revision surgery.

**Figure 4:**
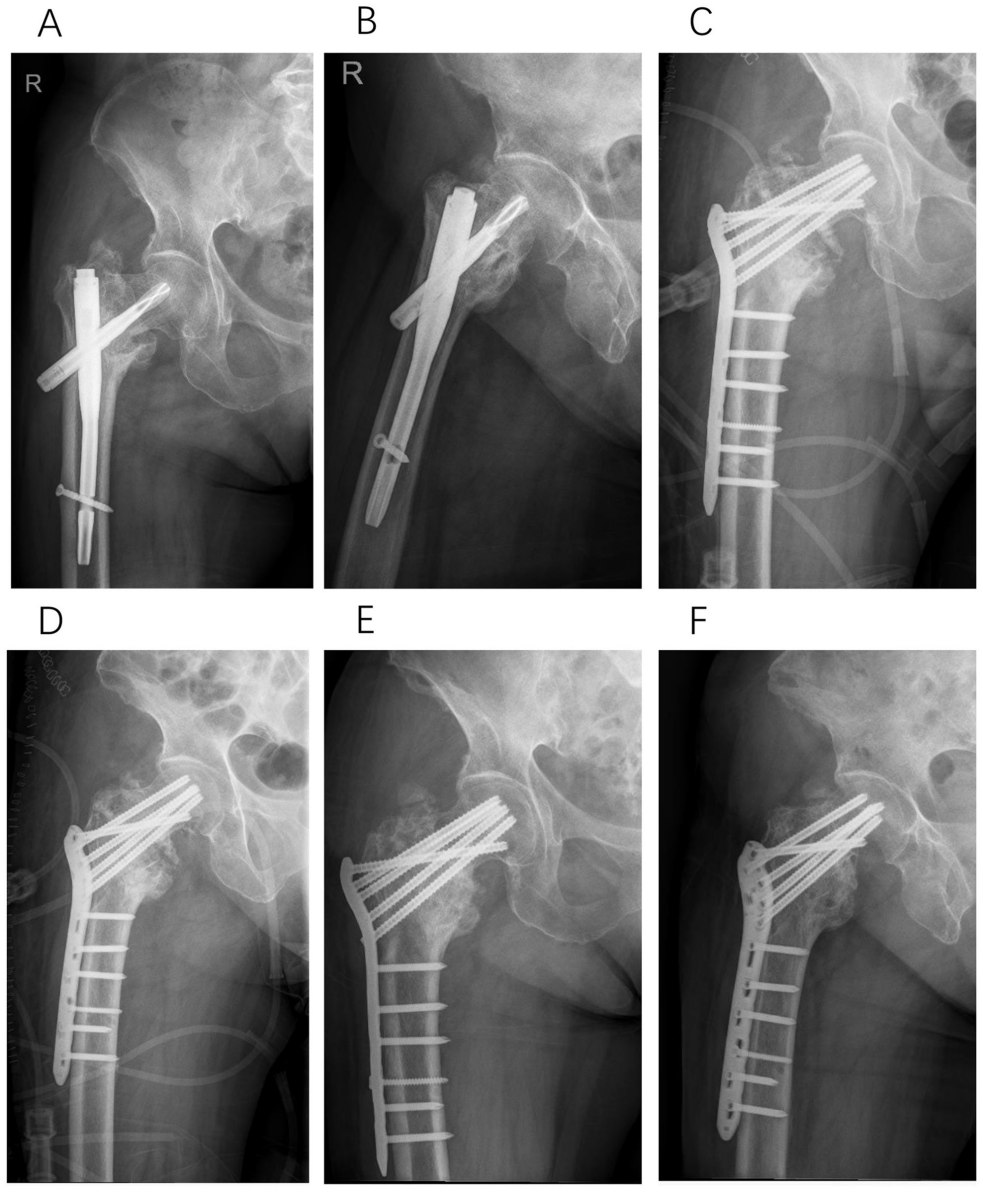
Type II-B, a 69-year-old male patient underwent PFNA fixation for a right intertrochanteric femoral fracture. Twelve months post-operatively, radiographic evaluation revealed coxa vara and non-union at the fracture site. In the subsequent revision surgery, we addressed the varus deformity through corrective osteotomy, performed structural bone grafting, and implemented plate internal fixation. Successful bony union was achieved 5 months following this revision. **(A, B)** Anteroposterior and lateral X-rays of the femur obtained 12 months after initial PFNA fixation. **(C, D)** Anteroposterior and lateral X-rays of the femur immediately following the revision surgery. **(E, F)** Successful bony union confirmed 5 months post-revision surgery.

**Figure 5:**
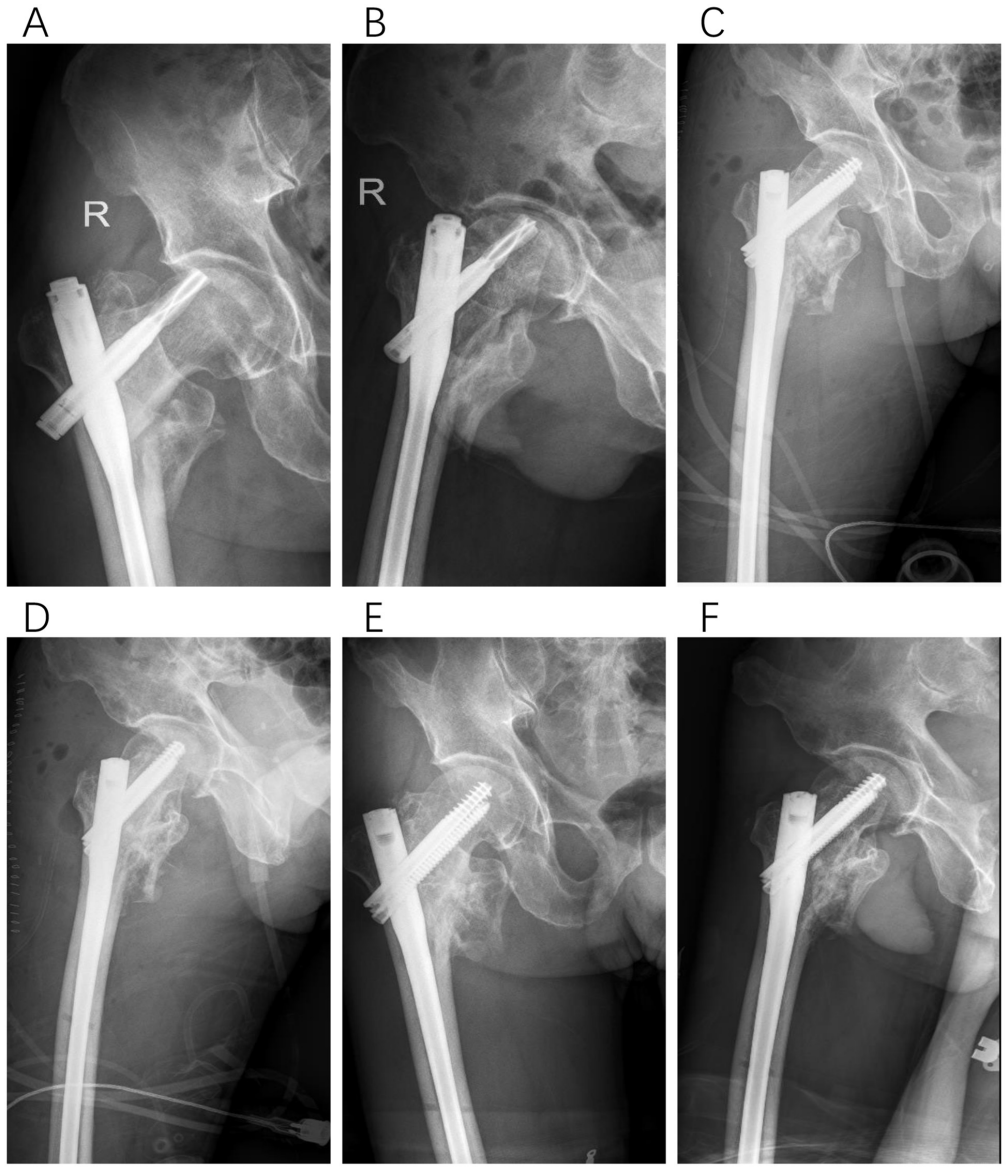
Type II-C, a 62-year-old female patient underwent PFNA fixation for a right intertrochanteric femoral fracture. Seven months post-operatively, radiographic evaluation revealed screw cutout through the femoral head. During the subsequent revision surgery, considering the relatively young age of the patient and minimal damage to the femoral head, we performed reduction, artificial bone grafting, and internal fixation with intramedullary nails. Successful bony union was achieved 6 months following this revision. **(A, B)** Anteroposterior and lateral X-rays of the femur obtained 7 months after initial PFNA fixation. **(C, D)** Anteroposterior and lateral X-rays of the femur immediately following the revision surgery. **(E, F)** Successful bony union confirmed 6 months post-revision surgery.

**Figure 6:**
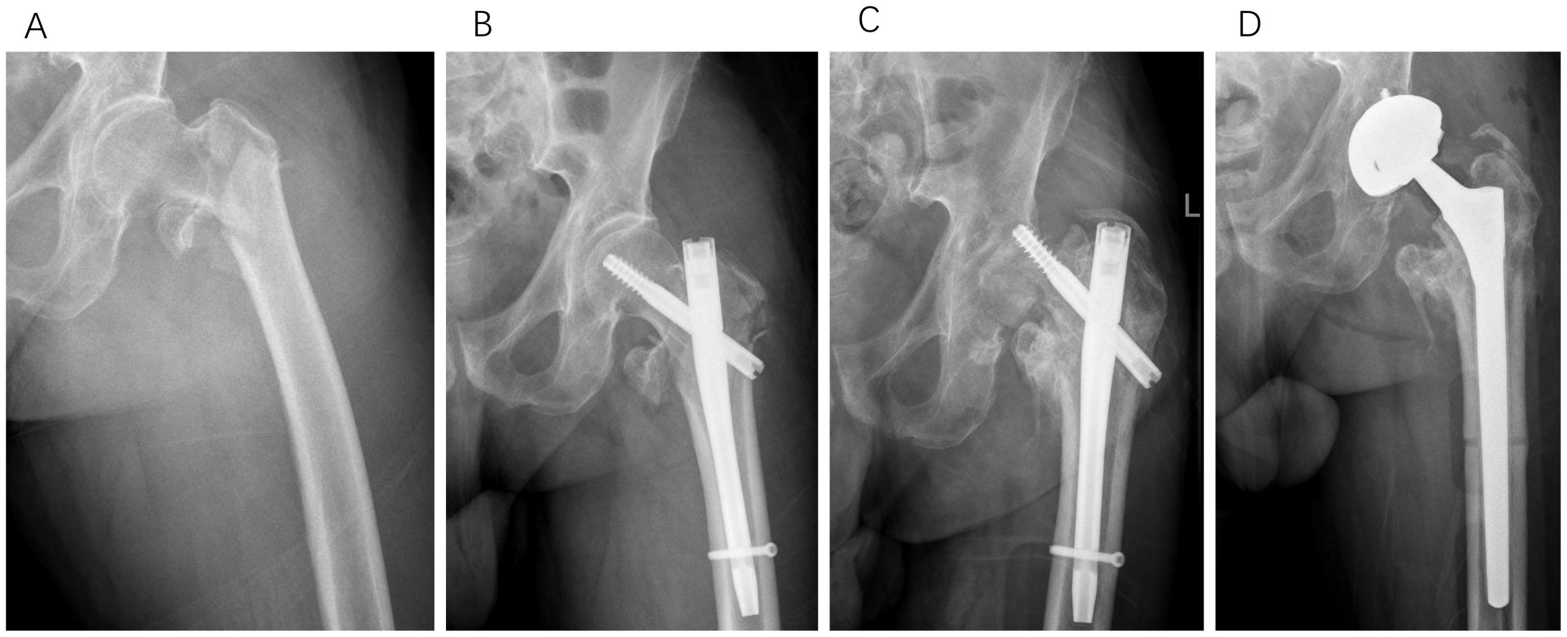
Type III, a 67-year-old male patient underwent Gamma nail fixation for a left intertrochanteric femoral fracture. Thirteen months post-operatively, radiographic evaluation revealed screw cutout and femoral head necrosis. In the subsequent revision surgery, we performed total hip arthroplasty. **(A)** Pre-operative anteroposterior X-ray of the injured hip. **(B)** Anteroposterior X-ray immediately following initial Gamma nail fixation. **(C)** Anteroposterior X-ray obtained 13 months after initial Gamma nail fixation. **(D)** Anteroposterior X-ray immediately following the revision surgery with total hip arthroplasty.

**Figure 7:**
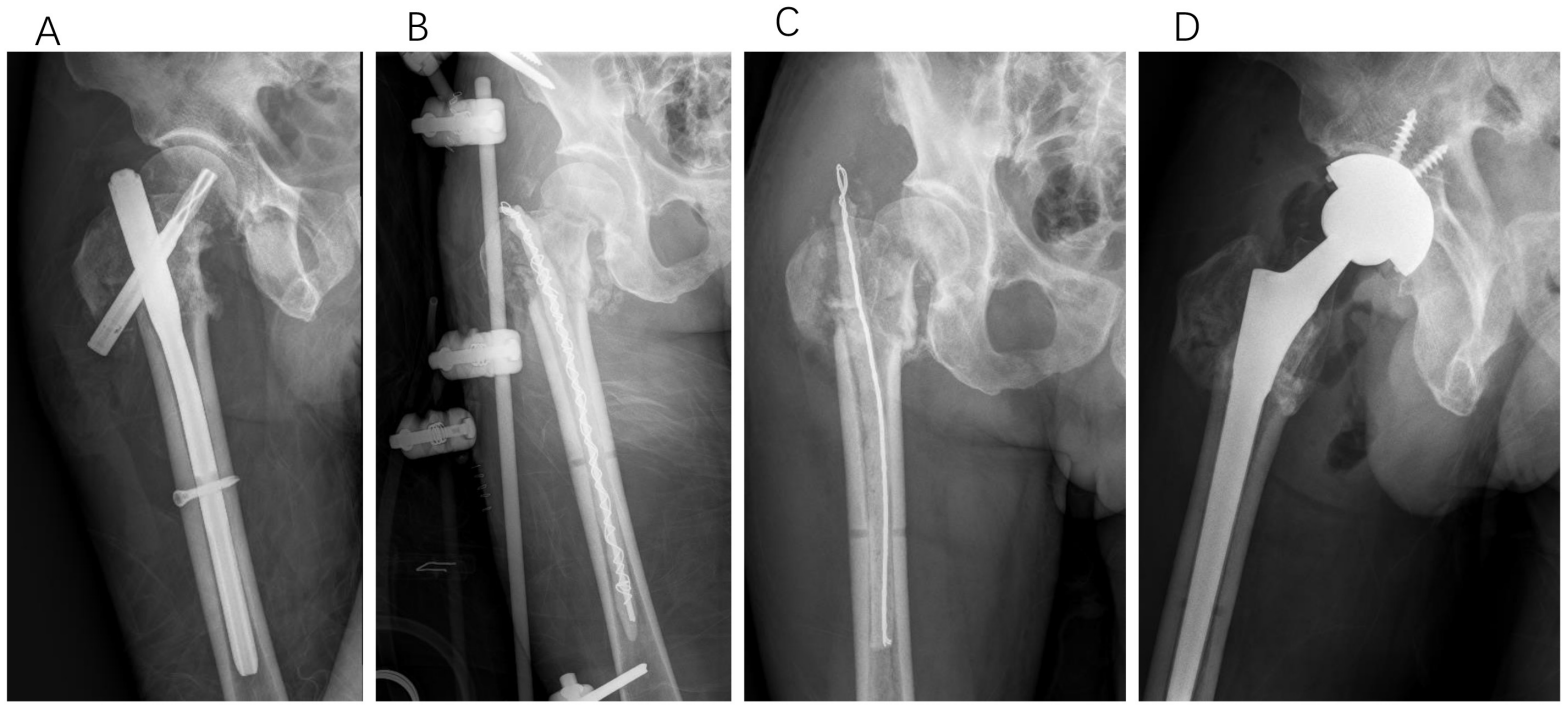
Type IV, a 65-year-old male patient underwent PFNA internal fixation for bilateral intertrochanteric femoral fractures. Post-operatively, the wound on the right side exhibited poor healing, necessitating revision surgery 5 months later. During the subsequent revision surgery, we performed extensive debridement, filled the medullary cavity with an antibiotic-impregnated cement rod, and provided temporary stabilization using an external fixator. After 2 months, a second debridement was conducted, and the antibiotic-impregnated cement rod was replaced. Following 4.5 months of treatment, the infection was successfully controlled. Due to the limited residual bone mass of the femoral head and the high risk of necrosis, total hip arthroplasty was ultimately performed. **(A)** Anteroposterior X-rays obtained 5 months after initial PFNA fixation. **(B)** Anteroposterior X-rays acquired immediately following the first revision surgery. **(C)** Anteroposterior X-rays acquired immediately following the second revision surgery. **(D)** Anteroposterior X-rays acquired immediately following the final revision surgery.

For each category, we formulated corresponding treatment strategies. For patients in group I, we opted for thorough debridement of the fracture site, supplemented with an appropriate amount of iliac bone grafting. Additional plates or thicker/longer intramedullary nails were added if necessary to enhance stability (Figure 2). For patients in group II, we resolved to remove the internal fixation devices, thoroughly debride the fracture sites, realign the bone fragments to restore an optimal neck-shaft angle and anteversion angle, and perform autologous iliac bone grafting as required (Figures 3–5). In cases where indicated, we employed large structural iliac bone grafts. A consistent revision strategy was applied to all Type II cases, where patients were stratified into three subgroups based on the primary mechanism of implant failure. Due to insufficient subgroup sample sizes, formal subgroup analyses were not conducted. In group II-C, “minimal femoral head damage” was defined radiographically as screw cut-out limited to cancellous bone, with no femoral head collapse, intact articular cartilage, and no acetabular involvement, allowing for joint-preserving revision surgery. For patients in group III, we removed the internal fixation device and performed either artificial femoral head replacement or total hip arthroplasty (Figure 6). For group IV, a minimum of two debridement procedures was required. The initial procedure involved removal of the internal implant, extensive debridement, and collection of five tissue samples from distinct anatomical sites—rather than purulent material—for microbiological culture, antibiotic susceptibility testing, and, when feasible, next-generation sequencing. Wound management included vacuum-assisted closure, and external fixation was applied to maintain fracture stability. A second procedure was typically performed approximately 1 week later. If the wound condition was satisfactory, an antibiotic-impregnated cement rod was implanted to fill the medullary cavity; otherwise, repeated extensive debridement was undertaken. Microbiological cultures were obtained during each debridement. After effective infection management, revision internal fixation or joint arthroplasty was performed (Figure 7).

### 2.4 Post-operative management

Routine post-operative care includes the administration of antibiotics for prophylactic anti-infection treatment and low-molecular-weight heparin to prevent lower-extremity venous thrombosis. On the first post-operative day, the patients initiated a regimen of ankle pump exercises and quadriceps muscle contractions to facilitate early mobilization and enhance recovery. Following joint replacement surgery, patients were encouraged to commence ambulation with the assistance of a walker immediately after the procedure. For patients with internal fixation, weight bearing on the unaffected limb was facilitated using bilateral crutches. In type IV revision groups, all patients received empirical intravenous antibiotic therapy (vancomycin + meropenem) immediately after the initial debridement. This was subsequently adjusted based on intraoperative tissue culture and sensitivity testing results. The standard regimen consisted of 2 weeks of intravenous antibiotics followed by 4 weeks of oral therapy. Follow-up evaluations were scheduled at 1, 2, 3, 6, 9, and 12 months post-operatively, followed by biannual assessments. At each visit, comprehensive functional and radiographic assessments were conducted. The Harris score (HSS) served as the key metric for evaluating functional outcomes.

### 2.5 Statistical analysis

Statistical analyses were performed using SPSS version 22.0 (IBM, Armonk, NY, USA). Differences among the four study groups were assessed by one-way ANOVA or the Kruskal-Wallis H test, followed by *post hoc* pairwise comparisons when overall significance was reached. The significance level was set at 0.05.

## 3 Results

### 3.1 General condition of patients

This study encompassed a total of 141 cases of failed internal fixation for femoral peri-trochanteric fractures, with a mean follow-up duration of 15.96 ± 5.61 months. The study cohort comprised 87 males and 54 females. Specifically, 96 cases of inter-trochanteric fractures were classified according to the Arbeitsgemeinschaft für Osteosynthesefragen/Orthopedic Trauma Association (AO/OTA) system: 28 type A1, 20 type A2, and 48 type A3 fractures. Additionally, the cohort included 45 cases of meticulously classified subtrochanteric fractures according to the Seinheimer system: 18 cases were type 2, 20 were type 3, six were type 4, and one was type 5 (Table 1).

**Table 1:** Patient demographics.

**Variables**	**Total (141)**	**I (45)**	**II (57)**	**III (32)**	**IV (7)**
Age (years)	60.34 ± 16.02	56.13 ± 14.32	57.42 ± 16.03	72.13 ± 13.00	58.86 ± 13.15
Males/females (*n*)	87/54	32/13	37/20	12/20	6/1
Affected side (right/left)	60/78	20/25	24/33	14/18	2/5
BMI (range), (kg/m^2^)	23.22 ± 3.38	24.39 ± 2.75	22.87 ± 3.24	22.65 ± 3.92	21.08 ± 3.31
Intertrochanteric fractures	96	19	40	32	5
**AO/OTA fracture type**, ***N*** **(%)**					
31A1	28 (29.2)	3 (15.8)	10 (25.0)	14 (43.7)	1 (20.0)
31A2	20 (20.8)	1 (5.3)	6 (15.0)	11 (34.4)	2 (40.0)
31A3	48 (50)	15 (78.9)	24 (60.0)	7 (21.9)	2 (40.0)
Subtrochanteric fractures	45	26	17	0	2
**Seinheimer fracture type**, ***N*** **(%)**					
Seinheimer 2	18 (40.0)	11 (42.3)	7 (41.2)	0	0
Seinheimer 3	20 (44.5)	9 (34.6)	9 (52.9)	0	2 (100)
Seinheimer 4	6 (13.3)	5 (19.2)	1 (5.9)	0	0
Seinheimer 5	1 (2.2)	1 (3.9)	0	0	0
**Fixation used**, ***N*** **(%)**					
Intramedullary nail	108 (76.6)	37 (82.2)	42 (73.7)	24 (75)	5 (71.4)
Single lag screw CMN	72 (51.1)	17 (37.8)	33 (57.9)	18 (56.2)	4 (57.1)
Dual lag screw CMN	26 (18.4)	14	6 (10.5)	6 (18.7)	0
RCN	10 (7.1)	6	3 (5.3)	0	1 (14.3)
DHS	3 (2.1)	0	0	3 (9.4)	0
Proximal locking plate	30 (21.3)	8 (17.8)	15 (26.3)	5 (15.6)	2 (28.6)

### 3.2 Implants information

The initial fixation methods employed for the included patients were as follows: 72 patients were treated with cephalomedullary nails (CMN) equipped with a single lag screw, 26 with cephalomedullary nails featuring dual lag screws, 10 with reconstruction nails (RCN), 30 with plate fixation, and three with a dynamic hip screw (DHS). Seven patients underwent revision surgery utilizing intramedullary nailing. Among these, six received a long cephalomedullary nail with dual lag screws, and one received a long reconstruction nail. Among the 45 patients with subtrochanteric fractures, the initial fixation method was intramedullary nailing in 35 cases and plate fixation in 10. Of the 35 initial intramedullary nailings, only three used short nails, while the remaining 32 used long nails. The revision strategy for subtrochanteric fractures mainly involved either augmenting the existing intramedullary nail with an auxiliary plate or completely removing the nail and transitioning to plate fixation. Arthroplasty was conducted as a revision procedure in 32 cases: 24 total hip arthroplasties (THA) and eight hemiarthroplasties (HA). Plate fixation was carried out as a revision procedure in 54 cases.

### 3.3 Clinical outcomes

**Figure 8:**
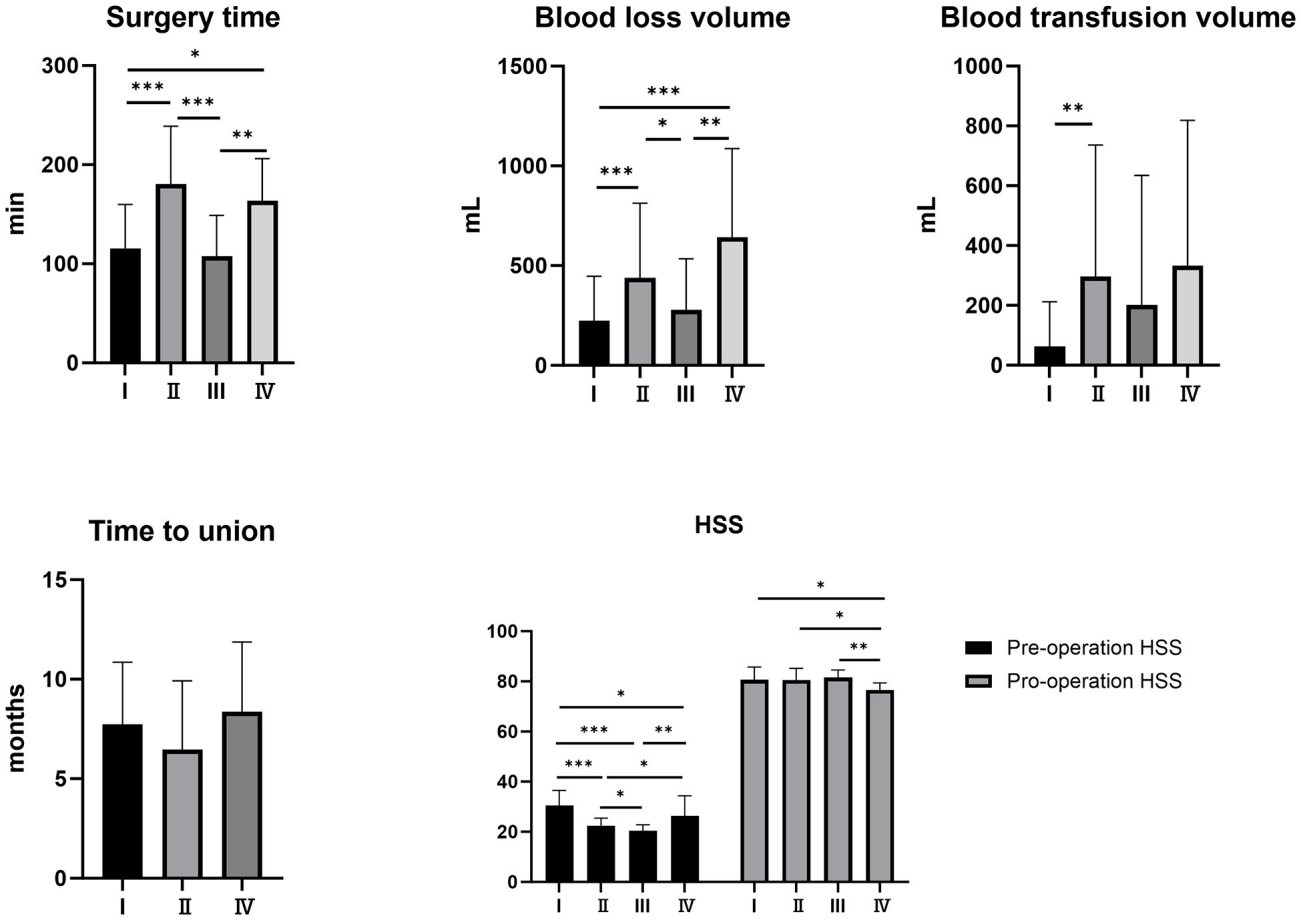
Comparison of clinical outcomes across the four classifications. *P < 0.05, **P < 0.01, ***P < 0.001.

In accordance with our revised classification, there were 45 cases of type I (including three cases involving simple bone grafting), 57 cases of type II, 32 cases of type III, and seven cases of type IV fractures. The mean age of all patients was 60.34 ± 16.02 years. The joint replacement group had a mean age of 72.13 ± 13.00 years, which was significantly higher than that of the revision internal fixation group (*P* < 0.05). The average duration of revision surgery was 143.06 ± 57.29 min. Specifically, the joint replacement group had an average operation time of 107.50 ± 41.40 min, which was significantly shorter compared to the type II and type IV revision groups (*P* < 0.01); however, no significant difference in operation time was observed between the joint replacement and type I revision groups. Regarding intraoperative blood loss and transfusion volume, the mean blood loss was 344.26 ± 335.43 ml, while the mean transfusion volume was 206.74 ± 495.95 ml. The type I revision and joint replacement groups had lower intraoperative blood loss than the type II and IV revision groups (*P* < 0.05). No significant differences in blood loss or transfusion volume were noted between the joint replacement and type I revision groups. The type I revision group exhibited a lower intraoperative blood transfusion volume than the type II revision group (*P* < 0.01). The mean duration from initial revision surgery to fracture healing was 7.08 ± 3.33 months. Among the groups, the type IV revision cohort exhibited the longest healing period of 8.38 ± 3.03 months; however, this difference was not statistically significant. As quantified by the HSS score, hip joint function improved from a pre-operative mean of 24.78 ± 6.08 to a post-operative mean of 80.59 ± 4.54. Notably, the post-operative HSS scores in the type IV revision group were significantly lower than those in the other three groups (*P* < 0.05). See details in Table 2 and Figure 8.

**Table 2:** Comparison of clinical outcomes across the four classifications.

**Variables**	**Total (141)**	**I (45)**	**II (57)**	**III (32)**	**IV (7)**
Surgery time (min)	143.06 ± 57.29	115.62 ± 44.33	180.45 ± 58.42	107.50 ± 41.40	163.57 ± 42.49
Blood loss volume (ml)	344.26 ± 335.43	223.18 ± 224.05	439.29 ± 374.25	277.59 ± 256.16	642.86 ± 443.55
Blood transfusion volume (ml)	206.74 ± 495.95 ml	62.50 ± 149.67	296.48 ± 440.13	201.48 ± 433.01	333.33 ± 485.34
Time to union (months)	7.08 ± 3.35	7.73 ± 3.12	6.47 ± 3.45	/	8.37 ± 3.50
Pre-operation harris scores	24.78 ± 6.08	30.58 ± 5.99	22.49 ± 2.97	20.37 ± 2.39	26.29 ± 8.10
Pro-operation harris scores	80.59 ± 4.54	80.62 ± 5.06	80.53 ± 4.71	81.53 ± 3.00	76.57 ± 2.82

## 4 Discussion

Hip fractures in elderly patients, often referred to as “the final fracture of one's life”, are associated with significant mortality, morbidity, and profound functional impairment (3). With an aging population, the number of hip fractures worldwide is expected to increase from 1.66 million in 1990 to an estimated 6.25 million by 2050, representing an annual growth rate of approximately 3% (12). Despite substantial improvements in patient outcomes achieved through timely surgical intervention, 6%−20% of patients continue to experience complications leading to internal fixation failure (10). Zhang et al. (15) conducted a retrospective analysis of 204 patients treated with proximal femoral nail anti-rotation II (PFNA II) for intertrochanteric fractures and identified 22 cases of internal fixation failure. This study revealed that factors, such as inadequate fracture reduction, advanced age, femoral neck reconstruction, and unstable fractures, were significantly associated with higher failure rates. Yamamoto et al. (16) noted that intramedullary malreduction of the anteromedial cortex was associated with failed internal fixation, emphasizing that immediate post-operative imaging alone was insufficient to predict internal fixation failure. Inui et al. (17) reported that inadequate anterior cortical reduction was significantly associated with screw cutout. Additionally, Konstantinidis et al. (18) highlighted that when bone mineral density falls below 250 mg/cm3, the risk of peri-trochanteric fracture fixation failure increases substantially. Similarly, Xu et al. (19) identified the Singh index as an independent predictor of internal fixation failure in peri-trochanteric fractures.

For orthopedic surgeons, selecting an appropriate revision strategy to minimize surgical trauma and enhance the success rate of operations is a critical clinical challenge in patients who have experienced failed primary surgeries for femoral trochanteric fractures (20). Key considerations in formulating these strategies include patient age, location of non-union (inter-trochanteric or sub-trochanteric), residual bone quality, and the condition of the internal implant. This study systematically analyzed 141 cases of failed internal fixation of femoral peri-trochanteric fractures, culminating in the development of a tailored revision strategy and workflow specifically designed to address the unique challenges associated with diverse types of initial treatment failures.

In this study, type I patients included those with the original implant that exhibited no signs of breakage or loosening and had a well-aligned position, accounting for approximately 32.6% of cases. Among the revision cases of sub-trochanteric fractures, 60% were classified as type I. The non-union in these patients may be attributed to insufficient blood supply or mechanical instability. Therefore, the primary approach to revision surgery involves autologous bone grafting, either alone or in combination with auxiliary plating. To enhance the probability of fracture union, we recommend employing bicortical plates and screws that traverse a minimum of six cortices (21). Three of the 46 type I patients in this study underwent simple autologous bone grafting, while the remaining 43 received combined treatment with auxiliary plates. During follow-up, the overall fracture healing rate was remarkably high, with only one patient failing to achieve union. The patient experienced plate failure and varus deformity, necessitating a subsequent revision according to the type II-B protocol. Ultimately, bony union was achieved after the intervention.

Type II failures were primarily attributed to mechanical instability, accounting for 39.7% of all cases. Among these, patients with type II-A malocclusion exhibit discontinuity or loosening of the original internal fixation devices. For these patients, our revision strategy involved removing the original internal fixation and replacing it with stronger devices, such as longer and thicker intramedullary nails, or substituting the locking plates with intramedullary nails/dynamic hip screws, in combination with autologous bone grafting. It is important to note that the replacement of the internal fixation can be technically challenging owing to differences in device design, particularly concerning the neck-shaft and valgus angles, which can significantly impact the biomechanical stability of the hip joint. Coxa vara is a common complication following peri-trochanteric fractures, often leading to hip and knee pain, limb length discrepancy, muscle imbalance, and abnormal gait (22, 23). For type II-B patients who not only have non-union but also suffer from coxa vara, our approach involves removing the original internal fixation, correcting the hip varus deformity, performing iliac structural bone grafting, and placing a robust internal fixation to ensure long-term stability and facilitate healing. Age (particularly < 65 years) and the extent of femoral head damage are the core criteria for considering joint preservation. In younger patients, if imaging meets all criteria for “minimal femoral head damage”, the case is classified as type II-C, and joint-preserving revision is strongly indicated. Conversely, if there is evidence of femoral head collapse, articular cartilage violation, or acetabular involvement, the case is classified as type III, necessitating arthroplasty. For younger patients, the indications for joint preservation can be appropriately liberalized. The recommended approach for type II-C includes removing the original internal fixation, correcting the hip varus deformity, and using a strong internal fixation along with autologous bone transplantation. This strategy aims to maintain joint integrity and restore function, especially in patients with high activity demands and a relatively preserved hip anatomy.

Type III patients exhibit significant femoral head and acetabular joint surface destruction. In managing this complex condition, it is essential to conduct a thorough and meticulous evaluation of the patient's age, functional demands, and the extent of damage to both the femoral head and acetabulum (24, 25). In the present study, arthroplasty was conducted as a revision procedure in 32 cases: 24 THA and eight HA. Among the HA cases, seven involved patients aged >80 years. The remaining patient was 62 years old; this individual was selected for HA due to poor general health status and significant comorbidities, including hearing impairment and hemiparesis. In general, HA may be an appropriate option for elderly patients with limited mobility and minimal acetabular damage. Conversely, THA is more suitable for patients with higher activity demands or significant acetabular joint surface damage.

Infection-related non-union is a significant cause of failure in peri-trochanteric fractures (13, 26). In our study, this condition accounted for 5.0% of cases. If the pre-operative white blood cell count, CRP, and ESR levels are elevated and intraoperative pathology reveals more than five neutrophils per high-power field, infection should be suspected (27). After confirming the diagnosis of infection, we removed the internal fixation device, performed thorough debridement, applied antibiotic-impregnated cement rods to fill the medullary cavity, and used external fixation to temporarily stabilize the fracture site. After the CRP level and ESR normalize, typically within 6 weeks, revision internal fixation surgery or joint arthroplasty is performed. The primary complications associated with type IV cases include persistent or recurrent infection, femoral head necrosis, bone defects, and joint dysfunction. In this study, one patient required a second debridement and cement rod exchange due to an uncontrolled infection following the initial antibiotic-impregnated cement rod implantation. Two patients required hip arthroplasty due to femoral head necrosis, whereas the other five patients achieved bony union. All seven patients exhibited varying degrees of bone defect. Post-revision functional outcomes, as evaluated by the HSS score, were the lowest among the other three groups.

This study systematically compared the healing times and clinical outcomes of different types of non-union fractures. The results demonstrated that type IV non-union exhibited significantly higher intraoperative blood loss than the other types. Regarding healing time, although type IV showed a longer duration than the other groups, this difference was not statistically significant, likely due to the relatively small sample size (*n* = 7) of type IV cases. Additionally, revision surgery for type II non-union was significantly longer, which may be attributed to the increased difficulty in repositioning old fractures. Furthermore, we evaluated the surgical conditions and post-operative function of patients who underwent joint replacement and internal fixation revision surgery. Patients in the joint replacement group were significantly older, had shorter surgery times, and experienced lower intraoperative blood loss than those with type II and type IV non-union; however, no significant differences were observed when compared to type I patients. Post-operative functional scores were not significantly different between patients who underwent joint replacement and those who underwent internal fixation revision surgery. These findings are consistent with prior research (11). Therefore, in our treatment protocol, young adults with mild femoral head injuries were classified as type II, with the objective of preserving hip joint function.

This study had some limitations that should be considered when interpreting the findings. First, as a retrospective analysis conducted at a single center, it is inherently susceptible to selection bias, which could affect the generalizability of our results. Second, the assessment of functional outcomes primarily relied on the HSS score, which was not performed under blinded conditions; this may have introduced observer bias. Third, despite our analysis, unmeasured confounding factors, such as detailed patient comorbidities, bone mineral density, and post-operative compliance, could have influenced both the risk of mechanical failure and the eventual clinical outcomes. Fourth, all patients in this study underwent bone grafting procedures, although some failures due to mechanical instability theoretically may not have required such interventions. Finally, given the relatively low incidence of this condition, the sample size was modest, potentially limiting the statistical power and robustness of our conclusions. Future research should benefit from larger-scale randomized controlled trials to further validate these findings.

## 5 Conclusion

Despite the relatively low failure rate of internal fixation of peri-trochanteric fractures, addressing such failures can be particularly challenging. It is crucial to carefully analyze the underlying causes and formulate precise individualized revision strategies. We categorized peri-trochanteric fracture internal fixation failures into four types based on age, presence of infection, condition of the femoral head and acetabulum, varus deformity, and implant loosening or rupture. We developed a corresponding treatment flowchart for each type. By adhering to our treatment protocol, all patients achieved satisfactory radiographic outcomes and functional recovery. Our treatment strategy provides a valuable reference for standardizing the revision of peri-trochanteric fractures.

## Data Availability

The original contributions presented in the study are included in the article/supplementary material, further inquiries can be directed to the corresponding author.
